# The contribution of the sphingosine 1-phosphate signaling pathway to chronic kidney diseases: recent findings and new perspectives

**DOI:** 10.1007/s00424-024-03029-5

**Published:** 2024-10-09

**Authors:** Stephanie Schwalm, Roxana Manaila, Anke Oftring, Liliana Schaefer, Stephan von Gunten, Josef Pfeilschifter

**Affiliations:** 1 Institut für Allgemeine Pharmakologie und Toxikologie, Goethe-Universität Frankfurt Am Main, Theodor-Stern-Kai 7, 60590 Frankfurt Am Main, Germany; 2 Institut für Pharmakologie, Universität Bern, Inselspital, INO-F, CH-3011 Bern, Switzerland

**Keywords:** Sphingosine kinase, Glomerulosclerosis, Diabetes mellitus, Inflammation, Fibrosis, TGF-β

## Abstract

Chronic kidney disease (CKD) is a multifactorial condition with diverse etiologies, such as diabetes mellitus, hypertension, and genetic disorders, often culminating in end-stage renal disease (ESRD). A hallmark of CKD progression is kidney fibrosis, characterized by the excessive accumulation of extracellular matrix components, for which there is currently no effective anti-fibrotic therapy. Recent literature highlights the critical role of sphingosine 1-phosphate (S1P) signaling in CKD pathogenesis and renal fibrosis. This review provides an in-depth analysis of the latest findings on S1P metabolism and signaling in renal fibrosis and in specific CKDs, including diabetic nephropathy (DN), lupus nephritis (LN), focal segmental glomerulosclerosis (FSGS), Fabry disease (FD), and IgA nephropathy (IgAN). Emerging studies underscore the therapeutic potential of modulating S1P signaling with receptor modulators and inhibitors, such as fingolimod (FTY720) and more selective agents like ozanimod and cenerimod. Additionally, the current knowledge about the effects of established kidney protective therapies such as glucocorticoids and SGLT2 and ACE inhibitors on S1P signaling will be summarized. Furthermore, the review highlights the potential role of S1P as a biomarker for disease progression in CKD models, particularly in Fabry disease and diabetic nephropathy. Advanced technologies, including spatial transcriptomics, are further refining our understanding of S1P’s role within specific kidney compartments. Collectively, these insights emphasize the need for continued research into S1P signaling pathways as promising targets for CKD treatment strategies.

## Introduction

### Overview: chronic kidney disease and fibrosis

Chronic kidney disease (CKD) is a multifactorial condition arising from various etiological factors. In the Western world, diabetes mellitus and hypertension are the leading contributors, followed by other causes such as inflammatory kidney diseases, genetic mutations, and drug-induced nephrotoxicity. Over prolonged periods of persistent irritation, these diverse disease pathways culminate in irreversible morphological alterations in the kidney, leading to organ dysfunction and even organ failure and end-stage renal disease (ESRD) [51, 60, 96, 122]. CKD is a major public health concern, affecting approximately 10–16% of the global population, thus imposing a significant socio-economic burden. Current therapeutic options are aiming primarily at slowing CKD progression or mitigating its complications and are of limited success. These strategies predominantly include glucocorticoids, renin–angiotensin–aldosterone system (RAAS) inhibitors, and sodium-glucose cotransporter-2 (SGLT2) inhibitors. However, despite these interventions, clinical outcomes remain suboptimal, as evidenced by dramatically rising global morbidity and mortality rates [29, 122].

Kidney fibrosis is a hallmark of CKD progression, characterized by the abnormal accumulation of extracellular matrix components. The fibrotic process shares similarities with normal wound healing, involving an inflammatory response that triggers the release of pro-inflammatory mediators, which promote the infiltration of immune cells such as neutrophils and macrophages into the damaged tissue. This cascade leads to the activation of resident fibroblasts, pericytes, and mesangial cells, which then differentiate into myofibroblasts, the primary producers of extracellular matrix. However, unlike in normal wound healing, fibrosis lacks the regulatory apoptotic elimination of myofibroblasts, leading to their persistent activity and the continuous production of fibrotic tissue. This pathological process can affect all compartments of the kidney, including the tubulointerstitial space, glomeruli (as seen in glomerulosclerosis), and the vasculature (as seen in arteriosclerosis), contributing to a progressive decline in renal function [78]. Despite these severe implications, there is currently no available anti-fibrotic therapy for CKD.

### S1P: biosynthesis and signaling pathways

Among the approximately 700 known sphingolipid metabolites, S1P stands out as one of the most extensively researched members. It is well-established that S1P significantly contributes to a range of physiological and pathophysiological cellular processes, including cell survival, proliferation, apoptosis, and migration. These cellular functions place S1P at the center of higher-order regulatory mechanisms such as immune responses, tumor development, angiogenesis, and fibrosis [46, 102].

S1P biosynthesis is a tightly regulated process mediated by two isoforms of sphingosine kinases (SphKs): SphK1 and SphK2. Although these enzymes generate the same product—S1P—in an ATP-dependent manner, they differ in substrate preferences, expression patterns, subcellular localization, and physiological roles. For instance, studies on knockout mice show that individual deletion of either SphK1 or SphK2 does not result in overt phenotypic abnormalities. However, the simultaneous deletion of both enzymes is embryonically lethal, highlighting their essential yet overlapping roles. These kinases control distinct subcellular pools of S1P, thereby influencing shared as well as unique biological pathways [3, 42, 70].

Interestingly, SphK1 is primarily cytoplasmic and translocates to the plasma membrane or nucleus upon activation by external stimuli, such as growth factors (e.g., PDGF and EGF), cytokines (e.g., TGF-β and TNF-α), hormones (e.g., angiotensin-II and insulin), and stress factors like hypoxia [91]. In contrast, SphK2 localizes to mitochondria and the endoplasmic reticulum and can shuttle in and out of the nucleus due to the presence of nuclear localization and export sequences [24, 67]. The genetic knockout of SphK1 reduces plasma S1P levels by approximately 50%, while Sphk2 knockout paradoxically increases plasma S1P levels, likely due to altered S1P clearance by the liver [53].

S1P can signal both intracellularly and extracellularly. While intracellular S1P targets such as TRAF2, prohibitin, and PPARγ remain somewhat controversial [91], extracellular S1P is actively secreted by transporters such as Spinster-2 (Spns2), major facilitator superfamily transporter 2b (Mfsd2b), and ABC transporters [33, 74, 119]. Spns2 knockout mice exhibit a 50% reduction in circulating S1P levels and an accumulation of S1P in tissues [73, 109]. Interestingly, these mice are protected in various models of inflammation and autoimmunity, suggesting that the distribution of S1P between intra- and extracellular compartments plays a key role in disease modulation [12, 25].

Once in circulation, S1P binds to specific chaperones—primarily ApoM (60%) in HDL and albumin (30%) in the lipoprotein-free fraction—ensuring its stable transport to target cells [89]. Extracellular S1P interacts with five G-protein-coupled receptors (S1PR1-5), activating downstream signaling pathways such as the ERK/MAPK, PI3K/Akt, and HIPPO/YAP pathways [110]. In the kidney, S1PR1, S1PR2, and S1PR3 are predominantly expressed, while recent findings highlight a pro-fibrotic role for S1PR5 in renal tissue [28]. The sphingosine analog fingolimod (FTY720; Gilenya®) has been a critical tool in exploring S1P signaling. Fingolimod is a broad-spectrum agonist for S1PR1, S1PR3, S1PR4, and S1PR5 but not S1PR2. It is a pro-drug, requiring phosphorylation by SphK2 for activation. Upon activation, fingolimod induces internalization and degradation of S1PR1, functionally antagonizing S1P/S1PR1 signaling. This mechanism traps T and B lymphocytes in secondary lymphoid organs, leading to peripheral lymphopenia, which is the basis for its use in treating multiple sclerosis [47].

Besides its transport into the extracellular space, cells can eliminate S1P through two primary pathways. It can be irreversibly cleaved by S1P lyase into hexadecenal and phosphoethanolamine, which are further metabolized into palmitoyl-CoA or utilized in phospholipid biosynthesis [55]. Alternatively, S1P can be dephosphorylated by S1P phosphatases, re-entering the sphingolipid metabolic cycle as sphingosine.

The specific distribution of S1P—regulated by transporters, chaperones, and metabolic enzymes—coupled with the distinct expression patterns of the five S1PRs, orchestrates its diverse physiological and pathological roles, including its involvement in kidney fibrogenesis. The importance of S1P in CKD and fibrotic processes in the kidney has been well documented [26, 46, 103]. This review focuses on summarizing the most recent insights into the role of S1P signaling in chronic fibrotic kidney disease.

## Role of S1P signaling in the fibrotic niche

Growing evidence suggests that tissue fibrosis originates within a specialized environment known as the fibrotic niche, where fibrotic lesions localize at distinct focal points rather than uniformly across the kidney parenchyma. In this niche, damaged tissue coexists with various non-parenchymal cell types, and different cellular states, which interact and cross-communicate. Spatial transcriptomic analyses of human kidneys have revealed that the fibrotic niche consists of mesenchymal cells, immune cells, and distinct tubular epithelial cells [62]. Fibrotic niches develop following tubular injury, recruiting immune cells that secrete profibrotic mediators, leading to myofibroblast activation—a central event in fibrogenesis. Interstitial myofibroblasts, characterized by elevated α-smooth muscle actin expression, play a key role in tissue repair. However, their persistent activation contributes to renal fibrosis, primarily through TGF-β/Smad signaling [36, 37].

**Fig. 1:**
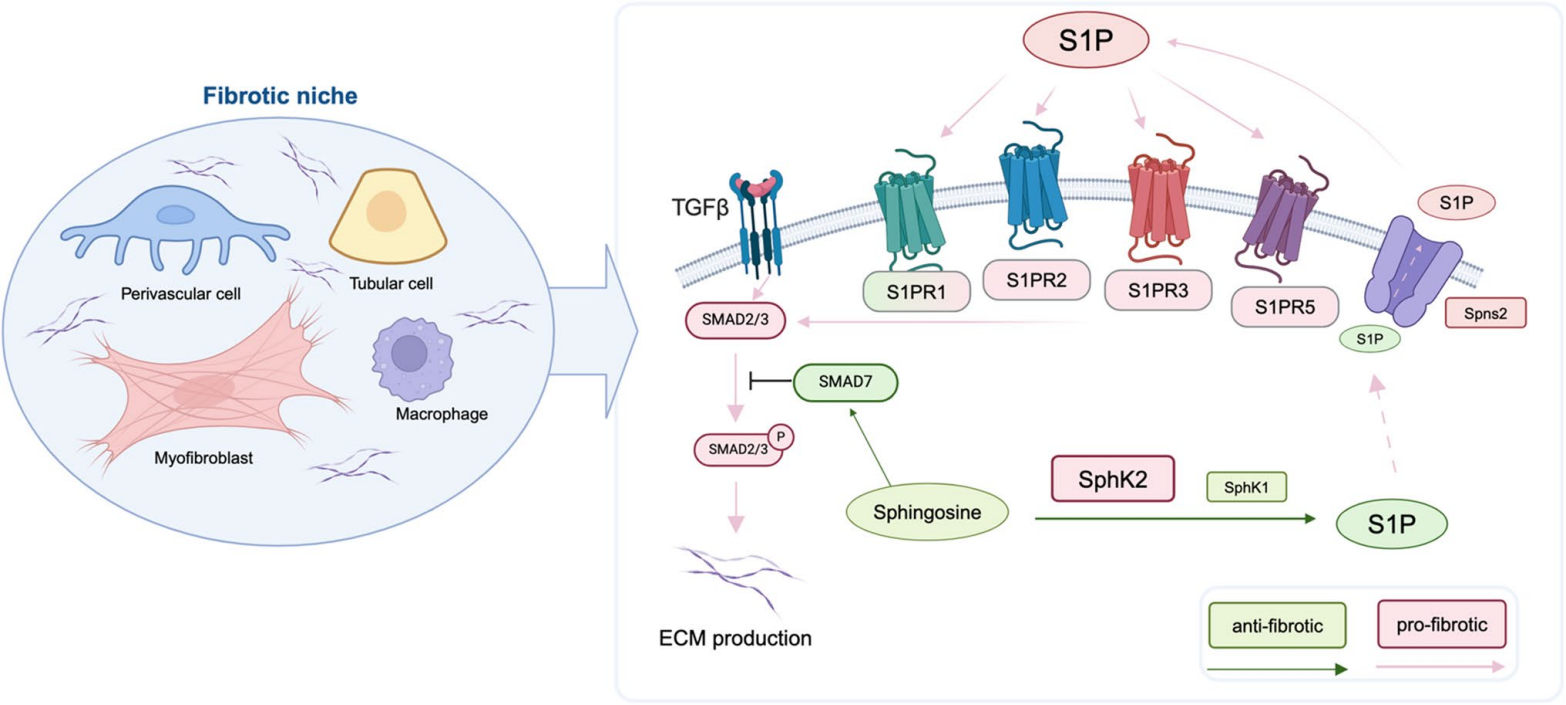
**Dual role of sphingosine 1-phosphate (S1P) signaling in kidney fibrosis.** This figure highlights the role of S1P metabolism and signaling in kidney fibrosis, emphasizing its dual effects within the fibrotic niche—a specialized microenvironment where various cell types, such as myofibroblasts, perivascular cells, tubular cells, and macrophages, drive fibrotic processes. S1P mediates either pro- or anti-fibrotic effects depending on its localization and interaction with specific S1P receptor subtypes (S1PR1, S1PR2, S1PR3, S1PR5) in renal cells. Red boxes and arrows illustrate pro-fibrotic actions. Intracellular S1P is transported to the exracellular space by Spinster-2 (Spns2). Extracellular S1P, through activation of S1PR1, S1PR2, S1PR3, and S1PR5, transactivates TGF-β signaling, leading to the phosphorylation of Smad2/3 and increased extracellular matrix (ECM) production, which drives the progression of kidney fibrosis. Green boxes and arrows represent anti-fibrotic actions. Intracellular S1P reduces renal fibrosis, and S1PR1 signaling on tubular cells has protective effects. The synthesis of S1P is mediated by sphingosine kinases (SphK1 and SphK2), which exert opposing effects on renal fibrosis: SphK1 is anti-fibrotic, while SphK2 is pro-fibrotic. Additionally, the S1P precursor, sphingosine, has been demonstrated to upregulate Smad7, which inhibits the phosphorylation of Smad2/3, thereby reducing ECM production and preventing fibrosis

S1P signaling plays a crucial role in fibrogenesis, as it can trans-activate the profibrotic TGF-β/Smad pathway through its receptors [36, 37]. Notably, intracellular S1P exhibits anti-fibrotic properties via mechanisms that remain unclear, while extracellular S1P generally promotes fibrosis [46, 90, 102, 103]. Interestingly, inhibiting extracellular S1P transport using the Spns2 inhibitor SLF1081851 [32] significantly attenuated the fibrotic response [114], highlighting the importance of S1P localization (Fig. 1).

Regarding the impact of the SphKs, most research has focused on SphK1, leaving SphK2 less explored. However, recent studies have identified SphK2 as a critical factor in myofibroblast activation and kidney fibrosis. Increased SphK2 expression has been observed in mouse models of kidney fibrosis and fibrotic kidney tissue from patients, confirming its pro-fibrotic role [7, 36, 100, 130]. SphK2 knockout mice show reduced tubulointerstitial inflammation and fibrosis in models such as unilateral ureteral obstruction (UUO) and folic acid-induced nephropathy. This protection is associated with increased expression of anti-fibrotic Interferon-γ [7], a rise in M2 macrophages [36], and reduced expression of AKT and STAT3 [130]. Additionally, our group found upregulation of Smad7, which inhibits the pro-fibrotic TGF-β/Smad pathway [100]. Through RNA sequencing and ChIP-Seq analysis, bone marrow stromal cell antigen-1 (Bst1/CD157) has been identified as a SphK2-regulated gene, playing a role in leukocyte recruitment. Of note, Bst1-deficient mice showed reduced renal fibrosis upon UUO [50].

Importantly, pharmacological inhibition of SphK2, using inhibitors such as ABC294046, SLM6031434, HWG-35D, and SLP120701, mimics the protective effects seen in SphK2 knockout mice [7, 36, 101, 130]. Conversely, a human SphK2 transgenic mouse line (hSphk2-tg) exhibited more severe UUO-induced fibrosis, further underscoring SphK2’s pro-fibrotic role in the kidney [100]. Recent studies using conditional mouse models indicate that perivascular cell-derived S1P synthesized by SphK2 contributes significantly to renal fibrosis. Deletion of SphK2 in perivascular cells led to decreased collagen deposition and myofibroblast activation in models of ischemia–reperfusion injury and folic acid nephropathy [114]. While deleting SphK2 in proximal tubular cells did not affect fibrosis levels in ischemia–reperfusion injury [114], reducing Spns2 expression in these cells counteracted TGF-β-induced upregulation of CTGF, fibronectin, and MCP-1 [12].

Perivascular S1P release, which activates S1PR1, triggers the production of pro-inflammatory cytokines and chemokines, promoting fibrosis [114]. However, S1PR1 signaling in endothelial and proximal tubular cells is beneficial, contributing to vascular stability and reducing inflammation [8, 9, 83]. In this context, circulating S1P bound to ApoM promotes protective vascular signaling through S1PR1. Aged mice with reduced ApoM and diminished S1PR1 signaling show increased susceptibility to renal fibrosis [23]. This underscores the dual role of S1PR1, where it exhibits both pro-inflammatory and anti-inflammatory effects, depending on cell type and context.

Treatment with fingolimod has reduced renal fibrosis in UUO and polycystic kidney disease models, largely through decreased expression of P-selectin and VCAM-1, reducing endothelial activation and leukocyte recruitment [2, 118]. In renal fibroblast cell lines, fingolimod suppressed TGF-β-stimulated αSMA expression and collagen synthesis via inhibition of Smad2/3 and PI3K/AKT/GSK3β pathways [42]. The agonistic and subsequent antagonistic effects of fingolimod on S1PR1 raise questions about which mode of action contributes to its anti-fibrotic effects. Moreover, the role of fingolimod in modulating S1PR3-5 requires further exploration. Recent G-protein-coupled receptor profiling revealed that S1PR3 is one of the most upregulated GPCRs in activated kidney fibroblasts in models of UUO, diabetes, adriamycin nephropathy, and folic acid nephropathy [52]. Furthermore, S1PR5 has emerged as a novel pro-fibrotic receptor, with increased expression in fibrotic kidney tissue in hydronephrosis patients [28]. Mice deficient in S1PR5 displayed reduced tissue damage and fibrosis, along with improved renal function in adenine-induced nephropathy [28]. However, further research is needed to decipher the precise mechanisms and interactions of S1PR5 in renal disease.

In conclusion, S1P signaling plays a complex, cell-type-specific role within the fibrotic niche, modulating both pro- and anti-fibrotic processes in kidney disease (Fig. 1). The regulation of S1P receptors and S1P localization is critical for determining the balance between inflammatory and fibrotic responses. Further research is essential to fully unravel the mechanisms by which S1P signaling drives fibrogenesis in CKD.

## S1P signaling in chronic kidney disease and disease models

**Fig. 2:**
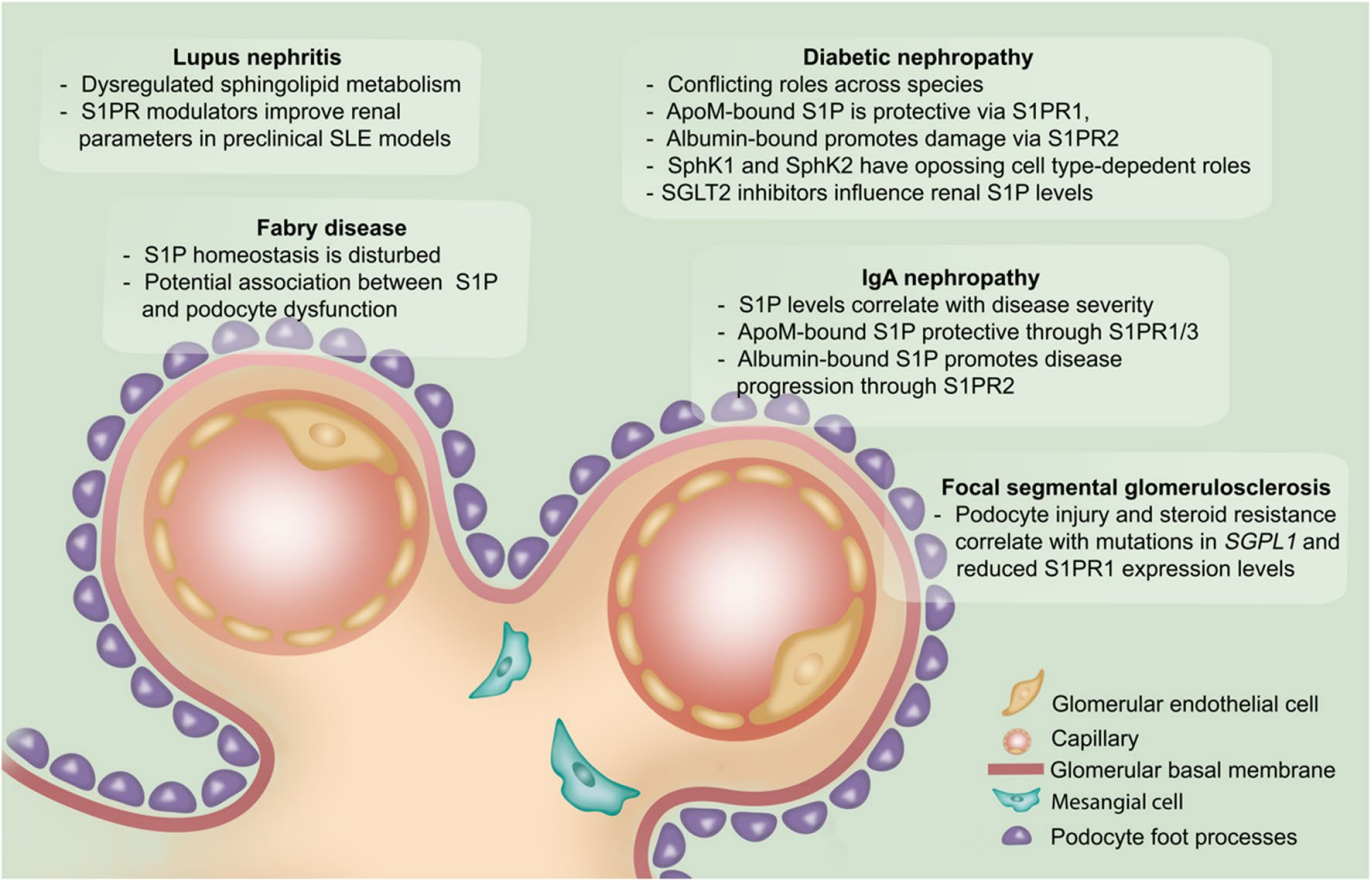
**S1P signaling in glomerular diseases: insights from recent studies.** This scheme highlights the role of sphingosine 1-phosphate (S1P) signaling in various glomerular diseases, incorporating findings from recent literature (2017 onwards). The figure outlines S1P’s involvement in lupus nephritis, diabetic nephropathy, Fabry disease, IgA nephropathy, and focal segmental glomerulosclerosis (FSGS). Key glomerular structures depicted include glomerular endothelial cells, the basal membrane, mesangial cells, and podocyte foot processes

The following part will delve deeper into the recent findings on the role of S1P signaling in specific chronic kidney diseases which are outlined in Fig. 2.

### Diabetic kidney disease

Diabetic kidney disease develops in approximately 40% of individuals with type 2 diabetes mellitus and remains the leading cause of ESRD worldwide. S1P signaling plays a complex and controversial role in the pathogenesis and progression of diabetic nephropathy (DN). In mouse models of diabetes, elevated S1P levels in plasma and renal tissue have been observed, contributing to renal inflammation, fibrosis, and tubular dysfunction, ultimately leading to DN [34, 75, 124]. However, these findings are not easily translatable to human studies, where diabetic patients often exhibit reduced circulating S1P levels, suggesting a potentially protective role for S1P in diabetes mellitus [47–49]. The divergent effects of S1P may stem from species differences, differential targeting of renal cell types, and the distinct carriers of circulating S1P. Among these carriers, ApoM-bound S1P is a known protective factor in the pathogenesis and progression of DN, with ApoM levels inversely correlating with disease progression. In streptozotocin (STZ)-induced diabetic mice, ApoM deletion worsened kidney damage, as evidenced by increased urinary albumin excretion, while ApoM overexpression reduced kidney dysfunction [56]. The protective effects of ApoM-bound S1P are believed to be mediated through biased agonism of S1PR1 [56], which enhances endothelial barrier integrity, and glomerular function, and reduces tubular inflammation and fibrosis [126, 127]. In contrast, albumin-bound S1P is associated with deleterious effects, facilitating S1PR2-mediated pro-inflammatory and pro-fibrotic signaling, thereby contributing to kidney damage [57]. These findings indicate that the activation of S1PR1 and S1PR2 confers antagonistic functions in fibrotic diabetic models in the kidney. Whereas S1PR1 reduces pro-fibrotic Smad3 activation and enhances vasoprotective eNOS phosphorylation, S1PR2 mediates opposite effects, enhancing Smad3 and reducing eNOS phosphorylation [56]. Additionally, S1PR2 promotes endothelial mesenchymal transition and impairs endothelial barrier function via a Wnt3a/RhoA/ROCK1/β-catenin signaling pathway [129].

Regarding S1PR3, single-cell RNA profiling indicates its expression in mesangial cells and may contribute to cell proliferation but its exact role in diabetes-induced kidney complications requires further evaluation [104].

In clinical studies, conflicting data exist regarding the relationship between plasma S1P levels and DN severity. For example, Bepkinar et al. reported lower plasma S1P levels in patients with macroalbuminuria compared to those without albuminuria, while Hammad et al. observed elevated S1P in patients with macroalbuminuria [10, 41]. These discrepancies may be attributable to differences in patient cohorts, blood sampling techniques, or S1P measurement methods [15]. Interestingly, extracellular vesicles isolated from the plasma of type 2 diabetic patients at progressive stages of DN contained increasing amounts of S1P, suggesting that vesicular S1P could serve as a biomarker for early DN diagnosis [77].

The role of S1P generated by the two sphingosine kinase isoforms, SphK1 and SphK2, in DN is another point of contention. Studies using genetic knockout models have shown that SphK2 depletion exerts glomerular and tubular renoprotective effects, reducing albuminuria and macrophage infiltration in the kidney [7, 49, 100]. In STZ-treated Sphk2-/- mice, the protection was associated with increased nephrin expression in podocytes, driven by the transcription factor WT1 [49]. However, the role of SphK1 remains unclear, with studies reporting opposing effects. In models induced by advanced glycation end products (AGE), SphK1 upregulation was required for FN-1 and ICAM-1 expression via activation of CK2α and NF-kB in mesangial cells, and SphK1 deletion resulted in fewer renal fibrotic lesions [18, 45]. Conversely, in podocytes, SphK1 appears protective, as Sphk1-deficient mice exhibited increased albuminuria and glomerular fibrosis in the STZ-induced diabetic model [93]. These findings highlight the need for further research to elucidate the cell type-specific roles of SphK1 in DN.

### Lupus nephritis

Systemic lupus erythematosus (SLE) is the archetypal multisystem autoimmune disease, characterized by excess autoantibody production to numerous cellular constituents, especially nucleic acids and nuclear proteins. Clinically, SLE can affect virtually any organ, with significant heterogeneity in both disease severity and organ involvement. Manifestations range from mild cases, such as those involving only the skin or joints, to life-threatening conditions with renal impairment, severe cytopenia, central nervous system disease, and increased thromboembolic events [44, 72]. Lupus nephritis (LN), which affects approximately one-third of SLE patients, significantly impacts survival [71]. The pathophysiology of LN is heterogeneous, involving both extrarenal and intrarenal mechanisms, and implicating both the innate and adaptive immune systems [59]. Despite improved understanding of LN pathogenesis, therapeutic advances remain limited, and the risk of kidney failure is still unacceptably high [79].

Emerging clinical evidence links dysregulated sphingolipid metabolism to SLE. Notably, patients with SLE exhibit altered circulating sphingolipid profiles. In a cross-sectional study of 103 SLE patients and 23 matched controls, Checa et al. observed significant increases in ceramides and decreases in S1P and sphingosine levels in SLE patients. Importantly, no correlation was found between plasma albumin and S1P levels, suggesting that S1P dysregulation occurs independently of the circulatory S1P carrier albumin [17]. In a longitudinal cohort (*n* = 22) from the same study, initiation of immunosuppressive therapy led to a reduction in ceramides and a significant increase in S1P levels. Furthermore, proteomic analysis revealed upregulation of ceramide synthase 5 and S1P lyase in SLE patients, suggesting that increased ceramide levels and decreased S1P might result from enhanced ceramide biosynthesis or S1P degradation [17]. Sphingolipid levels—except for hexosylceramide—did not differ between SLE patients with and without renal involvement, though larger cohorts are needed to confirm whether hexosylceramide could serve as a biomarker for active renal disease in SLE. In another study by Patyna et al., chain-length-specific ceramides, such as C24:1Cer, distinguished between SLE patients with and without renal involvement. Elevated plasma S1P levels were observed in LN patients compared to both healthy controls and SLE patients without LN; however, no difference was found between LN and non-LN SLE patients [81]. These findings highlight the complex role of S1P in LN, with larger studies required to elucidate its precise function. In juvenile SLE, while there was a trend toward elevated serum S1P levels, urine S1P concentrations did not differ between patients with renal and non-renal involvement, suggesting that the kidney may not be a direct source of S1P but rather responds to systemic S1P [121].

Studies on murine models of SLE further indicate alterations in sphingolipid metabolism, although the results are inconsistent. Snider et al., using high-performance liquid chromatography-tandem mass spectrometry (HPLC–MS/MS), observed elevated sphingosine and dihydro-S1P levels, but not S1P, in SLE mice [107]. Conversely, another study reported increased S1P levels in serum and kidneys of SLE mice measured by ELISA, with these elevations linked to M1-type macrophage accumulation via NLRP3 inflammasome activation through S1PR1 binding [117]. Given the established role of S1PR1 in immune cell recruitment during both acute and chronic inflammation, numerous preclinical and clinical studies support the use of S1PR agonists/modulators in treating immune-mediated conditions. The non-selective S1PR modulator fingolimod has previously been shown to be efficacious in reducing proteinuria and improving kidney histology in NZBWF1, BXSB, and MRL-lpr/lpr mouse models of SLE [4, 76]. The more selective amiselimod (S1PR1, S1PR4, S1PR5), ozanimod (S1PR1, S1PR5), and cenerimod (S1PR1) were also shown to confer efficacy and improve renal parameters in murine SLE [111, 113, 116]. Together, these studies provide evidence for the further development of the S1P/S1PR axis as a treatment strategy in LN.

### IgA nephropathy

IgA nephropathy (IgAN), first described in 1968 by Dr. Jean Berger, is the most prevalent form of glomerulonephritis worldwide, caused by the deposition of immune complexes containing galactose-deficient IgA1 in the glomeruli [95]. The diagnosis of IgAN is confirmed via kidney biopsy, which reveals characteristic immune deposits in the mesangial region. These deposits trigger mesangial cell proliferation and extracellular matrix deposition, leading to subsequent podocyte and tubulointerstitial injury. Over time, this pathological process progresses to ESRD in 30–40% of IgAN patients [58, 92].

Clinical presentations of IgAN vary significantly, ranging from mild microhematuria and proteinuria to severe macrohematuria and hypertension. Interestingly, these clinical manifestations correlate positively with urinary S1P levels [112]. Plasma S1P levels were found to significantly decrease in patients with low-grade proteinuria, compared to healthy controls, but increased back to control levels in patients with more severe kidney damage and higher proteinuria [112]. Investigations into the role of carrier-bound S1P in the progression of IgAN in HIGA mice (a model for IgA nephropathy) revealed that ApoM overexpression mitigated renal damage by suppressing mesangial expansion and fibrosis. Conversely, ApoM knockdown through siRNA exacerbated kidney injury, leading to increased proteinuria, a larger mesangial matrix area, and elevated expression of pro-fibrotic markers. The protective effects of ApoM were dependent on the bound S1P and inhibited by the S1PR1/3 antagonist VPC23019, but not by the S1PR2 antagonist JTE013, indicating the beneficial role of S1PR1/3-mediated signaling in renal protection [57]. In contrast, albumin-bound S1P contributed to the progression of renal injury by promoting mesangial proliferation and enhancing tubular expression of fibrotic markers through S1PR2 signaling. Although plasma S1P and ApoM levels were higher in HIGA mice compared to BALB control mice, the upregulation of renal S1PR2 likely overrode the protective S1PR1/3-mediated effects, resulting in a pro-fibrotic response [57].

S1P signaling has also been implicated in the formation of tertiary lymphoid organs (TLOs), which exacerbate kidney damage in IgAN. TLOs, which arise in response to chronic inflammation, are ectopic lymphoid structures found in non-lymphoid tissues and are composed of B and T lymphocytes, sometimes accompanied by lymphatic vessels and high endothelial venules [11]. In IgAN, the presence of TLOs is associated with disease progression and poor prognosis, and they have been identified as independent risk factors in the early stages of kidney injury [66]. S1P signaling plays a crucial role in the formation of TLOs, as fingolimod has been shown to inhibit TLO development and reduce the expression of pro-inflammatory cytokines and fibrotic markers, suggesting its potential as a therapeutic option for IgAN [66].

### Focal segmental glomerulosclerosis

Focal segmental glomerulosclerosis (FSGS) is not a specific disease, but a histopathological pattern characterized by localized scarring in the glomeruli. The etiologies of FSGS are diverse, encompassing genetic mutations, infections, medication toxicity, or idiopathic causes. Proteinuria is the hallmark symptom of FSGS, and the condition frequently progresses to CKD, glomerulosclerosis, and ESRD. FSGS accounts for 40% of nephrotic syndrome cases in adults and 20% in children in the USA and is identified in approximately 9% of all kidney biopsies in European adults [20]. Podocytes are the primary cellular target in FSGS, which undergo foot process effacement (PFPE) and depletion, leading to compromised glomerular filtration [21].

Recent research has highlighted the involvement of the S1P signaling pathway in genetic mutation-dependent FSGS. Sphingosine phosphate lyase insufficiency syndrome (SPLIS), first identified in 2017–2018, presents as steroid-resistant nephrotic syndrome and exhibits histopathological features similar to FSGS [19]. SPLIS is caused by recessive loss-of-function mutations in the Sgpl1 gene, which encodes S1P lyase (S1PL). S1PL is the only enzyme capable of irreversibly degrading S1P, serving as the exit point for sphingolipid metabolism. Its deficiency leads to excessive accumulation of S1P and other sphingolipids. In rodent models, partial Sgpl1 knockout or inhibition led to elevated intrarenal S1P levels and produced a phenotype resembling FSGS, with kidney sections displaying PFPE, chronic glomerular lesions, tubular degeneration, and interstitial fibrosis [99]. Mechanistically, podocytes are a primary target of elevated intracellular S1P due to S1PL depletion in the kidney. S1PL knockdown reduces the expression of nephrin and its regulatory transcription factor WT1, a reduction driven by elevated IL-6 levels, which in turn are triggered by S1P-mediated PKCδ activation [48]. Another study identified Crumbs homolog 2 (CRB2) gene mutations in a family with steroid-resistant nephrotic syndrome and FSGS. These mutations were linked to decreased expression and phosphorylation of S1PR1, which is known to protect against podocyte injury by upregulating podocyte-specific genes such as nephrin, podocin, and ZO-1. The reduction in S1PR1 likely contributes to the nephrotic phenotype, although the mechanism by which CRB2 mutations reduce S1PR1 expression remains unknown [125].

In an adriamycin-induced rat model of FSGS, serum S1P levels were found to inversely correlate with urinary protein content, suggesting its potential as a biomarker for advanced stages of nephrotic syndrome [61]. Additionally, in a mouse model of adriamycin-induced nephropathy, upregulation of SphK1 was associated with pro-inflammatory and pro-fibrotic responses. This effect was attenuated by the antioxidant resveratrol, which reduced renal inflammation and fibrosis by mitigating SphK1 induction [64].

### Fabry disease

Fabry disease (FD) is an X-linked sphingolipid lysosomal storage disorder caused by mutations in the GLA gene, which encodes for the enzyme α–galactosidase A (α–Gal A) [84]. Reduced activity of α-Gal A leads to the progressive accumulation of globotriaosylceramide (Gb3) and its deacetylated derivative globotriaosylsphingosine (lyso-Gb3), resulting in systemic manifestations, including CKD, cardiomyopathy, and stroke [1, 13, 22]. Since S1P is derived from sphingosine, which can be produced from the breakdown of glycosphingolipids, it is plausible that S1P homeostasis could be affected by the dysregulated sphingolipid catabolism in individuals suffering from an inherited lysosomal sphingolipid storage disease, such as FD. Furthermore, abnormalities in S1P may contribute to the pathophysiology of FD, although current data remain limited and are complicated by variations in analytical techniques used for quantification.

Burla et al. conducted a targeted lipidomics analysis on plasma and platelets in 15 FD patients (both males and females, treated with enzyme replacement therapy treated (ERT) or untreated) and 13 healthy controls [16]. Their study found that sphingoid 1-phosphate bases were decreased in plasma and serum but increased in platelets compared to healthy controls. Notably, most patients (11 out of 14) in this study were receiving ERT. Conversely, Brakch et al. found significantly elevated plasma S1P levels in 17 untreated FD patients compared with 17 healthy controls [14]. This discrepancy might be attributed to differences in treatment status or sample collection methods, as shown by Mirzaian et al., who demonstrated that sample handling can significantly impact S1P measurements [69]. In α-galactosidase A-deficient Fabry mice, no differences in plasma S1P levels were observed compared to controls. However, a twofold increase in renal S1P levels was reported, which is of interest given the podocyte dysfunction characteristic of Fabry nephropathy and the above-mentioned established link between increased S1P levels and severe forms of congenital nephrotic syndrome (SPLIS) [65]. While larger cohorts and more mechanistic studies are needed, these data suggest that S1P could serve as a potential diagnostic and prognostic biomarker in FD. Moreover, both preclinical and clinical data, although still limited, point toward an association between S1P and cardiovascular remodeling in FD. S1P treatment in mice led to Fabry-like hypertrophic cardiomyopathy, and in Fabry patients, plasma S1P levels positively correlated with common carotid artery intima-media thickness and left-ventricular mass index [14]. Similarly, S1P levels were elevated in non-classic Fabry patients, with a moderate positive correlation between S1P and interventricular septum thickness [14]. Both cardiomyocytes and renal cells share similar sensitivity to S1PR-mediated signaling pathways, contributing to pathological changes in hypertrophy and fibrosis, which underscores the systemic nature of sphingolipid dysregulation in Fabry disease. Hence, further studies of S1P metabolism would be worth the effort in FD patients, since many S1PR modulators are available.

## Contribution of S1P signaling on established kidney protective therapeutics

This chapter summarizes the effects of established treatment strategies for CKD, including glucocorticoids, sodium-glucose co-transporter 2 inhibitors (SGLT2i), angiotensin-converting enzyme inhibitors (ACEi), and angiotensin II receptor blockers (ARB) on the S1P pathway since an increasing amount of data suggest that the efficacy of these treatments may be partially mediated through the modulation of S1P levels or S1P signaling.

Glucocorticoids like dexamethasone, still a first-line therapy option for many kidney diseases, exert anti-inflammatory, immunosuppressive, and nephroprotective effects [87]. Dexamethasone differentially regulates the expression and activity of SphKs; it upregulates SphK1, which protects mesangial cells from stress-induced apoptosis [30], and downregulates SphK2 expression in podocytes. SphK2 downregulation not only enhances cellular sphingosine levels but also increases WT1 and nephrin expressions, suggesting that the nephroprotective effects of dexamethasone might be mediated through the downregulation of SphK2—a finding particularly beneficial in diabetic nephropathy and other glomerular kidney diseases [49]. These effects are mediated by the glucocorticoid receptor, as evidenced by their reversibility with the glucocorticoid receptor antagonist RU-486 (mifepristone). Additionally, similar to the findings in mesangial cells, there is a transient increase in SphK1 and a downregulation of S1PR1 in podocytes, which may contribute to nephroprotection [49].

SGLT2 inhibitors like empagliflozin (Jardiance®), approved in 2023 for CKD treatment in Europe, significantly reduce CKD progression by decreasing renal glucose reabsorption [43]. In a rat diabetes model, empagliflozin significantly decreased the elevated renal S1P content observed after disease induction, an effect not seen in the plasma, liver, or heart [82]. This suggests that the anti-inflammatory and anti-fibrotic effects of SGLT2 inhibitors in the kidney [115, 128] may indirectly involve reduced S1P signaling mechanisms. Furthermore, dapagliflozin has been shown to increase ApoM expression in mice, which mediates S1P-dependent endothelial barrier protection [94].

CKD often presents with an activated renin–angiotensin–aldosterone system (RAAS), and angiotensin II, via the Angiotensin type 1 receptor (AT1), contributes to inflammatory cell recruitment and fibrotic marker expression in the kidney. Hence, ACEi and ARB are effectively used to reduce hypertension and offer anti-fibrotic properties [98]. Nevertheless, there is limited literature on their impact on S1P levels and the responsible enzymes. In a pilot study involving 45 patients, aimed at identifying plasma markers predictive of responsiveness to treatment with the ACEi lisinopril, elevated levels of S1P and sphingosine were observed in responders compared to non-responders [108]. These findings suggest that the blood pressure-lowering effects of lisinopril may be partially mediated by an increase in circulating S1P levels. Another recent pilot study with 34 patients showed that circulating S1P levels increased after three months of treatment with ACEi but not with ARB treatment [86], indicating that the effects of ACEi on S1P are due to ACE blockade rather than reduced AT1 activation. These observations necessitate further investigation in larger cohorts and the identification of the involved mechanisms. Furthermore, a possible involvement of the S1P signaling pathway in the anti-fibrotic effect of ACEi and ARBs urgently needs to be investigated.

The reported findings highlight the complex interactions of these drugs with the S1P signaling pathway, offering insights into their mechanisms of action beyond their primary uses. Further studies are needed to detail these interactions and their clinical implications. Understanding these interactions may be crucial for optimizing therapeutic approaches and enhancing patient outcomes in CKD.

## Therapeutic options for S1P signaling in CKD and future perspectives

Despite some conflicting data, increasing evidence suggests that targeting specific components of S1P metabolism and signaling could serve as a promising therapeutic strategy for CKD and a summary of pharmacological modulators targeting S1P metabolism and signaling in the kidney is found in Table 1. Notably, the inhibition of SphK2 is considered attractive due to its established pro-fibrotic role in CKD. Opaganib, (3-(4-chlorophenyl)-N-(pyridin-4-ylmethyl)-1-adamantanecarboxamide, hydrochloride salt; ABC294640), an orally active, putatively selective SphK2 inhibitor with anti-proliferative, anti-inflammatory and anti-viral properties, is currently under investigation in clinical trials for conditions such as gastrointestinal acute radiation syndrome, COVID-19, and cholangiocarcinoma [106]. This compound has also demonstrated potential in reducing kidney damage and inflammation in acute kidney injury models and UUO-induced renal fibrosis in mice, suggesting its viability for clinical trials in patients with CKD and renal fibrosis. However, the specificity of Opaganib for SphK2 is debatable; some studies report a high in vitro IC_50_ value of 50 µM [31], while others find it completely ineffective at inhibiting recombinant SphK2 [88]. Moreover, in Sphk2-deficient renal mesangial cells, opaganib significantly reduced proliferation, indicating potential off-target effects [104]. Additional targets for opaganib that have been identified include dihydroceramide desaturase [68], glucosylceramide synthase [106], 3-ketodihydrosphingosine reductase [88], and the estrogen receptor [5, 68], further complicating its specificity profile. Given these challenges, newer and more potent SphK2 inhibitors such as SLM6031434, HWG-35D, and SLP120701 with lower IC_50_ values in the low µM or nM range are emerging as more promising candidates [54, 80, 105]. These have shown beneficial effects in models of kidney disease [36, 101]. However, caution is warranted when interpreting enzyme function in disease processes based solely on pharmacological modulators, as off-target effects can contribute to observed outcomes.

**Table 1:** Summary of pharmacological modulators targeting S1P metabolizing enzymes, transporters, and signaling in kidney diseases and kidney cells (2017–present)

Target	Modulator	Model/stimulus	Tissue/cell type	Key findings	References
SphK1	PF543 (inhibitor)	UUO	Mouse kidney, tubular epithelial cells	PF543 exacerbates renal fibrosis and inhibits autophagy	[27]
		-	Renal fibroblasts	PF543 in Sphk2^−/−^ cells reduces upregulated HIF2α and EPO production	[39]
SphK2	Opaganib (ABC294640, inhibitor)	UUO	Renal interstitial fibroblasts	Opaganib prevents renal fibrosis	[130]
		Cisplatin-induced nephropathy	Mouse kidney, renal tubular cells	Opaganib protects against cisplatin-induced kidney injury, suppresses inflammatory markers and apoptosis	[123]
	SLM6031434 (inhibitor)	UUO	Mouse kidney, renal fibroblasts	Sphk2 inhibition ameliorates kidney fibrosis via upregulation of Smad7	[101]
		-	Perivascular cells	SLM6031434 suppresses inflammatory signaling	[114]
		-	Podocytes	SLM6031434 protects against podocyte dysfunction	[49]
	HWG-35D (inhibitor)	UUO	Renal interstitial fibroblasts	HWG-35D prevents renal fibrosis	[130]
	SLP120701 (inhibitor)	UUO	Macrophages	SLP120701 prevents renal fibrosis and alters macrophage polarization	[35]
Spns2	SLF1081851 (inhibitor)	Unilateral IRI	Mouse kidney, perivascular cells	SLF1081851 reduces inflammatory signaling and ameliorates kidney fibrosis	[114]
S1PR1,3,4,5	Fingolimod (agonist, S1PR1 funct. antagonist)	Unilateral IRI	Mouse kidney	Fingolimod does not protect renal fibrosis	[114]
		IRI	Rat kidney	Fingolimod protects against IRI and improves renal antioxidant capacity	[6]
		Lupus nephritis (MRL/lpr mice)	Mouse kidney, macrophage	Fingolimod reduces M1 macrophage polarization, NLRP3 activation and nephritis symptoms	[117]
		Polycystic kidney disease models	Mouse kidney	Fingolimod improves disease progression via restoration of PKCζ activity	[2]
		Polycystic kidney disease (Cy/ + Han:SPRD rats)	Rat kidney	Fingolimod delays disease progression by attenuating NF-κB and STAT3 activation	[63]
		IgAN	Mouse kidney	Fingolimod prevents tertiary lymphoid organ formation, ameliorates renal fibrosis, and reduces inflammation	[66]
		AngII-induced KI model	Rat kidney, podocytes	Fingolimod reduces AngII-induced renal lesions, inflammatory cytokines, and protects podocytes against S1P-induced damage	[112]
		IRI	Mouse kidney, dendritic cells	Ex vivo fingolimod-treated dendritic cells mediate S1PR1-dependent protection in IRI	[97]
S1PR1, 4, 5	Amiselimod (MT-1303, agonist, S1PR1 funct. antagonist)	LN (NZBWF1, MRL/lpr)	Mouse kidney	Amiselimod reduces renal T/B cell infiltration, mesangial expansion, and glomerular sclerosis	[113]
S1PR1, 5	Ozanimod (RPC1063, agonist, S1PR1 funct. antagonist)	LN (NZBWF1 model)	Mouse kidney	Ozanimod reduces mesangial expansion, immune cell infiltration, and fibrosis	[116]
S1PR1	Cenerimod (funct. antagonist)	LN (MRL/lpr)	Mouse kidney	Cenerimod reduces proteinuria, renal T cell infiltration, and renal damage	[111]
S1PR1, S1PR3	VPC23019 (antagonist)	DN (STZ-induced)	Mouse kidney, podocytes	VPC23019 reverses ApoM overexpression-mediated renal protection	[56]
		IgAN (HIGA mice)	Mouse kidney, proximal tubular cells, mesangial cells	VPC23019 enhances kidney damage and reverses ApoM-mediated renal protection	[57]
	NIBR-0213 (antagonist)		Renal interstitial fibroblasts	NIBR-0213 reverses S1P-stimulated EPO production	[40]
	TY-52156 (antagonist)		Renal interstitial fibroblasts	TY-52156 reverses S1P-stimulated EPO production	[40]
S1PR2	JTE013 (antagonist)	DN (STZ-induced)	Mouse kidney, podocytes	JTE013 reduces proteinuria and fibrosis; does not affect ApoM overexpression protection	[56]
		IgAN (HIGA mice)	Mouse kidney, proximal tubular cells, mesangial cells	JTE013 mitigates kidney fibrosis, Smad3 activation and reverses albumin-bound S1P-induced fibrosis in tubular cells	[57]
		High glucose	Human glomerular endothelial cells	JTE013 blocks high glucose-mediated glomerular endothelial cell dysfunction	[120]
			Renal interstitial fibroblasts	JTE013 has no effect on S1P-stimulated Erythropoietin production	[40]

*UUO* unilateral ureteral obstruction, *IRI* ischemia-reperfusion injury, *DN* diabetic nephropathy, *IgAN* IgA nephropathy, *AngII* angiotensin II, *LN* lupus nephritis, *STZ* streptozotocin, *S1P* sphingosine 1-phosphate, *SphK1/2* sphingosine kinase ½, *S1PR1-5* sphingosine-1-phosphate receptors 1- 5, *EPO* erythropoietin, *HIF2α* hypoxia-inducible factor 2 alpha, *PKCζ* protein kinase C zeta, *NF-κB*: nuclear factor kappa B

Modulating S1PRs also presents a promising therapeutic approach for CKD. The administration of the non-selective S1PR modulator fingolimod has demonstrated beneficial outcomes in numerous rodent models of renal damage and fibrosis, enhancing renal function, exerting anti-inflammatory effects, and reducing fibrosis (reviewed in [46]). While fingolimod’s protective effects are partly attributed to the reduction in infiltrating lymphocytes via functional antagonism at S1PR1, additional peripheral mechanisms, independent of lymphopenia, are likely involved. The contribution of distinct S1PRs responsible for fingolimod’s renal protection, particularly the roles of S1PR3 and S1PR5, remains to be fully elucidated. Newer S1PR modulators, such as siponimod (S1PR1, S1PR5), ozanimod (S1PR1, S1PR5), and cenerimod (S1PR1), offer advances over fingolimod by providing more selective receptor targeting, improved pharmacokinetic profiles, and potentially reduced side effects. These compounds reduce the likelihood of off-target effects, such as bradycardia and liver toxicity, seen with fingolimod. As mentioned in the lupus nephritis section, ozanimod dose-dependently reduced proteinuria and alleviated mesangial expansion and interstitial fibrosis in the NZBWF1 mouse model of SLE [116]. And cenerimod, currently in a phase 3 clinical trial for systemic SLE (NCT06475742), has demonstrated efficacy in reducing T cell infiltration into the kidney, lowering plasma anti-dsDNA antibodies, and ameliorating tissue damage and proteinuria in the MRL/lpr mouse model of lupus nephritis [111].

In contrast to the compounds with functional antagonistic properties for S1PR1, SAR247799 is a novel selective S1PR1 biased agonist. SAR247799 preferentially activates G-protein signaling over β-arrestin and internalization pathways, thereby sustaining S1PR1 activation without receptor desensitization or inducing lymphopenia [85]. Given the bifunctional and cell-type-specific roles of S1PR1 signaling, exploring compounds like SAR247799 in renal diseases may prove beneficial by mimicking protective circulatory ApoM-bound S1P signaling while avoiding pro-inflammatory and pro-fibrotic S1PR1 signaling in the kidney.

Although some roles of S1PR2, S1PR3, and S1PR5 in CKD have already been characterized, further investigation using selective inhibitors is necessary to deepen our understanding of their specific contributions to disease progression in various CKD models. Additionally, Spns2 inhibitors show promise as therapeutic agents by potentially enhancing intracellular anti-fibrotic actions while reducing extracellular pro-fibrotic S1P activities.

The complexity of S1P metabolism and signaling is further compounded by the existence of several S1P variants with differing sphingoid base chain lengths. Besides the predominant variant d18:1 S1P, endogenous levels of d16:1 S1P and d20:1 S1P are also produced with a supposedly differential S1PR affinity [38]. Notably, d16:1 S1P has been implicated in mediating induction of the pro-fibrotic CTGF via activation of S1PR2 in renal cell carcinoma [37]. The occurrence and potential production of different S1P variants in renal inflammation and renal fibrosis are completely unknown and merit further investigation to understand their specific roles and impacts.

Taken together, further elucidation of S1P signaling pathways and their diverse roles in renal pathophysiology, through targeted therapeutic intervention and detailed study of S1P variants, holds the promise of uncovering novel treatment strategies that can effectively address the complexities of renal inflammation and fibrosis in CKD. Additionally, emerging technologies, such as spatial transcriptomics and advanced imaging techniques, are enabling a more refined spatial characterization of S1P signaling in CKD and kidney fibrosis, allowing for precise mapping of S1PR signaling within specific disease-related regions and cell types of the kidney. These advancements are crucial for identifying localized therapeutic targets and understanding the microenvironmental impacts of S1P signaling dynamics.

## Data Availability

No datasets were generated or analyzed during the current study.
